# Sacubitril/valsartan inhibits obesity-associated diastolic dysfunction through suppression of ventricular-vascular stiffness

**DOI:** 10.1186/s12933-021-01270-1

**Published:** 2021-04-21

**Authors:** Annayya R. Aroor, Srinivas Mummidi, Juan Carlos Lopez-Alvarenga, Nitin Das, Javad Habibi, Guanghong Jia, Guido Lastra, Bysani Chandrasekar, Vincent G. DeMarco

**Affiliations:** 1 Diabetes and Cardiovascular Center, University of Missouri School of Medicine, Columbia, MO USA; 2 Division of Endocrinology and Metabolism, Department of Medicine, University of Missouri-Columbia School of Medicine, D110, DC043.0 One Hospital Dr, Columbia, MO 65212 USA; 3 Research Service, Harry S. Truman Memorial Veterans Hospital, Columbia, MO USA; 4 South Texas Diabetes and Obesity Institute, Department of Human Genetics, School of Medicine, University of Texas Rio Grande Valley, Edinburg, TX USA; 5 Department of Cardiothoracic Surgery, University of Texas Health Science Center, San Antonio, TX USA; 6 Division of Cardiovascular Medicine, Department of Medicine, University of Missouri-Columbia School of Medicine, One Hospital Dr, Columbia, MO 65212 USA; 7 Dalton Cardiovascular Research Center, University of Missouri, Columbia, MO USA; 8 Department of Medical Pharmacology and Physiology, University of Missouri, Columbia, MO USA

**Keywords:** Obesity, Diabetes, Diastolic dysfunction, Neprilysin inhibition

## Abstract

**Objective:**

Cardiac diastolic dysfunction (DD) and arterial stiffness are early manifestations of obesity-associated prediabetes, and both serve as risk factors for the development of heart failure with preserved ejection fraction (HFpEF). Since the incidence of DD and arterial stiffness are increasing worldwide due to exponential growth in obesity, an effective treatment is urgently needed to blunt their development and progression. Here we investigated whether the combination of an inhibitor of neprilysin (sacubitril), a natriuretic peptide-degrading enzyme, and an angiotensin II type 1 receptor blocker (valsartan), suppresses DD and arterial stiffness in an animal model of prediabetes more effectively than valsartan monotherapy.

**Methods:**

Sixteen-week-old male Zucker Obese rats (ZO; n = 64) were assigned randomly to 4 different groups: Group 1: saline control (ZOC); Group 2: sacubitril/valsartan (sac/val; 68 mg•kg^−1^•day^−1^; ZOSV); Group 3: valsartan (31 mg•kg^−1^•day^−1^; ZOV) and Group 4: hydralazine, an anti-hypertensive drug (30 mg•kg^−1^•day^−1^; ZOH). Six Zucker Lean (ZL) rats that received saline only (Group 5) served as lean controls (ZLC). Drugs were administered daily for 10 weeks by oral gavage.

**Results:**

Sac/val improved echocardiographic parameters of impaired left ventricular (LV) stiffness in untreated ZO rats, without altering the amount of food consumed or body weight gained. In addition to improving DD, sac/val decreased aortic stiffness and reversed impairment in nitric oxide-induced vascular relaxation in ZO rats. However, sac/val had no impact on LV hypertrophy. Notably, sac/val was more effective than val in ameliorating DD. Although, hydralazine was as effective as sac/val in improving these parameters, it adversely affected LV mass index. Further, cytokine array revealed distinct effects of sac/val, including marked suppression of Notch-1 by both valsartan and sac/val, suggesting that cardiovascular protection afforded by both share some common mechanisms; however, sac/val, but not val, increased IL-4, which is increasingly recognized for its cardiovascular protection, possibly contributing, in part, to more favorable effects of sac/val over val alone in improving obesity-associated DD.

**Conclusions:**

These studies suggest that sac/val is superior to val in reversing obesity-associated DD. It is an effective drug combination to blunt progression of asymptomatic DD and vascular stiffness to HFpEF development in a preclinical model of obesity-associated prediabetes.

## Introduction

Heart failure (HF) with normal or preserved ejection fraction (HFpEF) represents a unique pathophysiological phenotype which is distinct from HF with reduced ejection fraction (HFrEF). In this regard, asymptomatic diastolic dysfunction (or preclinical DD) is one of the earliest manifestations during the progression of HFpEF in obesity-associated heart disease in both adults and children [1–3]. Moreover, the prevalence of DD is high in the general population and its incidence is further increased in individuals with early phase obesity-associated type 2 diabetes [4, 5]. In addition to low grade inflammation and intrinsic structural and functional abnormalities, vascular stiffness also contributes to DD development and progression to HFpEF in obese diabetic individuals [5–9].

There are no evidence-based therapies to treat HFpEF, and therefore therapeutic strategies are urgently needed to improve cardiovascular outcomes, including DD [10]. In this regard, dysregulation of the natriuretic peptide (NP) system and inappropriate activation of the Renin–Angiotensin–Aldosterone System (RAAS) contribute to the development of obesity-associated DD in the absence of significant impairment in EF, a marker of systolic dysfunction [13–18]. Although high doses of recombinant BNP, *e.g*., Nesiritide, are used in the management of acute cardiac dysfunction, their use in treating chronic HF is limited due to adverse hemodynamic consequences and a requirement for intravenous administration [11–13]. Inhibition of the RAAS system is considered as one of the therapeutic options for the management of DD, however, the long-term administration of angiotensin receptor blockers (ARBs) is associated with aldosterone escape [14, 15]. To circumvent these side-effects, a new class of drug containing the combination of sacubitril, a neprilysin inhibitor that blocks the degradation of NPs, and valsartan, an ARB, is increasingly recognized as an ideal combination to manage HFrEF [16, 17]. The rationale for combining valsartan with sacubitril is to overcome the deleterious effects of sacubitril-induced Ang II accumulation [12, 18–20].

A recent clinical trial reported that sacubitril-valsartan (sac/val) did not significantly lower rates of hospitalizations for HF and death from cardiovascular disease among patients with established HFpEF [21]. However, few studies have examined the effects of sac/val to blunt the severity of DD in pre- and early diabetic states of obesity-related cardiomyopathy. Recently, sac/val was shown to be superior to val at preventing the onset of DD in a preclinical model of diet-induced obesity [22], however, it is unknown whether sac/val can treat established DD in the setting of obesity. Furthermore, the immunological responses to sac/val have not been extensively investigated in these models.

Insulin resistant Zucker Obese (ZO) rats with established DD are extensively used as a genetic model for diet-induced obesity. A leptin receptor mutation in the ZO rat prevents hypothalamic binding of leptin resulting in hyperphagia leading to severe obesity and progression to early-stage type 2 diabetes. Moreover, the development of DD with insulin resistance at an early age in ZO rats also mimics the DD seen in obese young adolescents [3]. We have previously utilized ZO rats to elucidate the cellular and molecular mechanisms underlying reversal of obesity-associated DD by several therapeutics, including a beta blocker [23], a Dipeptidyl peptidase-4 (DPP-4) inhibitor, and a mineralocorticoid receptor blocker [24, 25]. Recently, we reported the beneficial effects of the combination of sac/val (LCZ696) in the treatment of early kidney injury in ZO rats [16]. In the present investigation, we investigated whether a ten-week treatment with sac/val could ameliorate progression of an already established abnormal cardiac phenotype in ZO rats. Moreover, we evaluated the efficacy of sac/val relative to val monotherapy. We have also analyzed changes in multiple cytokines using a cytokine array to identify novel potential targets for precision-based therapy. Herein, we report that sac/val, used as a treatment strategy, reduced the severity of DD in obese prediabetic ZO rats, and this was associated with increased IL-4 levels.

## Methods

### Animals

Sixty-four male Zucker Obese (ZO) and six age-matched Zucker Lean (ZL) rats were purchased from Charles River Laboratories (Wilmington, MA), and housed in a 12 h light/dark cycled room. Animals were cared for in accordance with the National Institutes of Health guidelines. All procedures were approved and performed in accordance with the Subcommittee for Animal Safety at the Harry S Truman Veterans Memorial Hospital and the Institutional Animal Care and Use Committee of the University of Missouri. All ZO rats were weighed prior to the start of the experiment and randomly distributed into four treatment groups so that each group had a similar mean body weight. Beginning at 16 weeks of age, ZO rats received sac/val (ZOSV; 68 mg•kg^−1^•day^−1^), val (ZOV; 31 mg•kg^−1^•day^−1^), hydralazine (ZOH; 30 mg•kg^−1^•day^−1^) or saline (ZOC) once daily for 10 weeks by gavage. Rats were gavaged at a similar time each morning (6:00–7:00 am central standard time). Guidelines for dosing rats with sac/val and val were provided by Novartis (document #RD-2016-00069). Further, we determined that 30 mg/kg/day hydralazine was the most appropriate dose based on a preliminary telemetric BP study evaluating BP responses to 10 and 30 mg/kg/day doses. In a recent report, we also reported the use of these drugs at a similar dose [16]. Body weights were measured weekly thereafter until the end of the experiment (26 weeks of age). Untreated age-matched male ZL rats served as lean controls (ZLC). Six rats were removed from the study due to complications associated with oral gavage. Following sacrifice epidydimal and retroperitoneal fat pad masses were excised and weighed and results were reported previously [16].

### Telemetric blood pressure monitoring

Under isoflurane anesthesia (2% isoflurane in a stream of O_2_), a subset of 13 week-old ZO rats (n = 16) were implanted with an abdominal aorta catheter attached to a radio transmitter (TA11PA-C40; Data Sciences International, St Paul, Minnesota), as previously described [16, 26]. After a 3 week recovery, both systolic (SBP) and diastolic blood pressures (DBP) were monitored in 300 s bins every 15 min for two 12 h light and two 12 h dark cycles (sampling rate, 1000 Hz), and telemetry data were analyzed post hoc. Monitoring periods ended 2 days prior to and approximately 3, 5, 7 and 9 weeks after treatment began. One rat was removed from the study prior to the start of BP monitoring due to complications from transmitter implantation surgery. We have previously reported mean arterial pressures in the same rats used in this study [16].

### Ultrasound assessment of aortic and cardiac function

Doppler ultrasound (Vevo 2100, Fujifilms, Visualsonics, Toronto, ON, Canada) studies were performed at the Small Animal Ultrasound Imaging Center at the Harry S Truman VA Research Center on isoflurane-anesthetized rats (1.75–2% in 100% oxygen stream) near study end utilizing an MS250 (13–24 MHz) echo probe. Rats were placed on a heated platform to maintain body temperature at 37 °C and heart rate of 400 to 450 bpm. In vivo aortic stiffness was evaluated by the transit time method to determine aortic pulse wave velocity (PWV), as previously described [27]. Briefly, PWV was calculated as the difference in arrival times of a Doppler pulse wave at two locations along the aorta at a fixed distance. Pulse wave arrival times are measured as the time from the peak of the ECG R-wave to the leading foot of the pulse wave at which time velocity begins to rise at the start of systole. The measured distance between the two locations along the aorta is divided by the difference in arrival times and is expressed in m/s. Velocity waveforms were acquired at the aortic arch followed immediately by measurement at the distal descending aorta a known distance from the aortic arch. Next, two-dimensional echocardiograms were performed in the apical four chamber view. In pulse wave (PW) Doppler mode, peak early (E) and late (A) diastolic blood flow velocities were obtained at the level of the mitral inflow stream just proximal to the mitral leaflets. From the PW spectra we determined isovolumic relaxation time (IVRT), isovolumic contraction time (IVCT) and ejection times, parameters needed to calculate the myocardial performance index (MPI), also known as the Tei index. MPI was calculated as the sum of isovolumic contraction and relaxation times divided by ejection time. B- and M-mode images of the left ventricle and septum in short axis view were acquired at the level of the papillary muscles. Left ventricular anterior and posterior wall thicknesses at end systole (LVAWTs and LVPWTs) and diastole (LVAWTd and LVPWTd), luminal diameters (LVIDs and LVIDd) and ejection fraction (EF) were determined offline in M-mode. Next, Tissue Doppler Imaging (TDI) was performed in the apical four chamber view by placing a sample volume at the septal annulus to acquire early (e’) and late (a’) septal annular velocities. Using a modified parasternal long axis view of the left ventricle and aortic root we determined stroke volume (SV) and cardiac output (CO). A sample volume was placed in the aortic outflow tract to obtain blood velocity spectra in Doppler PW mode. Offline calculation of the velocity time integral of PW traces and measurement of the maximum diameter of the ascending aorta acquired in B-mode were used to calculate SV and CO using onboard software. Parameters were assessed using an average of three heart beats from two to three different spectra, and calculations were made in accordance with the American Society of Echocardiography guidelines as well as specific guidelines for rodent echocardiography. All data were acquired and analyzed offline by a single blinded observer. Although we had previously reported an abnormal diastolic phenotype in young male ZO rats (24, 25 and 29), herein we performed cardiac ultrasound on ten randomly selected ZO rats prior to the beginning of the treatment period (15 weeks old) in order to determine whether these rats have an abnormal pre-existing cardiac phenotype (Table 1).

**Table 1:** Ultrasound derived cardiac function and structure parameters of Zucker Obese rats prior to the ten-week treatment period (N = 10; ZOC_/pre_) and following the ten-week treatment period (N = 6; ZOC_/post_)

Parameter sample size	ZOC_/pre_ (10)	ZOC_/post_ (6)
Body weight (g)	560 ± 7	752 ± 17*
Heart weight (mg)	NA	1429 ± 62
HW/TL (mg mm^−1^)	NA	18.1 ± 0.5
Systolic parameters		
Ejection fraction (%)	82 ± 2	76 ± 2*
Cardiac output (ml min^−1^)	302 ± 56	270 ± 29
Stroke volume (µl)	828 ± 164	762 ± 90
IVCT (ms)	12.5 ± 0.9	15.5 ± 0.6
s’ (mm sec^−1^)	43 ± 1	42 ± 3
Ejection time (ms)	65 ± 3	71 ± 2
LVETI	695 ± 22	685 ± 17
Systolic time (ms)	99 ± 3	112 ± 3*
Diastolic parameters		
E, (mm sec^−1^)	921 ± 49	1117 ± 126
A, (mm sec^−1^) late	828 ± 60	1036 ± 127
E/A ratio	1.06 ± 0.05	1.07 ± 0.08
e’, (mm sec^−1^)	36 ± 2	52 ± 6*
a’, (mm sec^−1^)	54 ± 5	69 ± 10
e’/a’ ratio	0.69 ± 0.06	0.76 ± 0.05
E/e’ (LV filling pressure)	24.5 ± 1.0	22.7 ± 2.6
Diastolic stiffness (E/e’/LVIDd)	3.26 ± 0.10	2.68 ± 0.29
IVRT (ms)	20.8 ± 0.9	25.8 ± 1.4*
MPI	0.54 ± 0.04	0.58 ± 0.01
Structural parameters		
LA/Ao ratio	1.51 ± 0.06	1.38 ± 0.11
LV Mass (mg)	926 ± 44	1067 ± 19*
AWTd (mm)	2.12 ± 0.09	2.07 ± 0.05
PWTd (mm)	1.87 ± 0.09	1.83 ± 0.11
Relative wall thickness	0.53 ± 0.03	0.46 ± 0.03
LVIDd (mm)	7.55 ± 0.11	8.45 ± 0.21*
LVIDs (mm)	3.60 ± 0.16	4.44 ± 0.16*

Values are mean ± SE, (sample sizes shown in parentheses). *p < 0.05 vs ZOC_/pre_. *HW* Heart weight, *tibia length* tibia length, *LV* left ventricle, *e’* early septal wall velocity during diastole, *a’* late septal wall velocity during diastole, *s’* peak septal wall velocity during systole, *E* early mitral flow velocity, *A* late mitral flow velocity, *E/E’ LV* filling pressure, *IVRT* isovolumic relaxation time; IVCT isovolumic contraction time; MPI myocardial performance index, *LVETI LV* ejection time index, *LA* left atrium diameter, *Ao* aorta diameter, *AWTd* anterior wall thickness at end diastole; *PWTd* posterior wall thickness at end diastole, *LVIDd LV* inner diameter at end diastole, *LVIDs LV* inner diameter at end systole

### Ex vivo aortic reactivity by wire myography

Vasomotor responses of aortae were examined as previously described [27, 28]. Briefly, a 2 mm segment of thoracic aorta, collected immediately after euthanasia, was placed in ice-cold physiological salt solution (PSS) containing (in mM): 119 NaCl, 4.7 KCl, 2.5 CaCl, 1.18 KH_2_PO_4_, 1.17 MgSO_4_, 0.027 EDTA, 5.5 glucose, and 25 NaHCO_3_, pH 7.4. Aortic contractile state was ascertained using 80 mM KCl. Initially, aortas were preconstricted with U46619 (100 nM), a thromboxane A2 mimetic. Relaxation responses of arterial rings to acetylcholine (1 nM to 100 μM) were assessed by cumulative addition of agonist to the vessel bath. At the end of each experiment, the PSS bath solution was replaced with Ca^2+^-free PSS to determine maximal passive diameter. Aortic dilator responses are presented as percent maximal relaxation.

### Ex vivo endothelial cell (EC) stiffness by atomic force microscopy (AFM)

The stiffness of EC, measured as the force exerted by a stylus probe on the luminal surface of aortic explants, was measured by a nano-indentation technique utilizing AFM, as previously described by us [27]. Briefly, a 2 mm ring of the thoracic aorta was isolated from rats to assess the stiffness of EC. The aortic ring was opened longitudinally, and the adventitial surface of each explant was fastened to a glass cover slip using Cell-Tak cell tissue adhesive so that we had en face access to the EC surface for placement of the AFM stylus. Stiffness of the EC surface was estimated by placing the stylus at approximately 15 random locations along the EC surface of an explant and determining the average EC stiffness for that aorta.

### Quantification of myocardial interstitial and periarterial fibrosis

Two mm-thick slices of the LV free wall were fixed in paraformaldehyde, embedded in paraffin, sectioned at five microns and stained for collagens using Picro Sirius Red (PSR), as previously described [29]. For each animal, an average estimate of interstitial fibrosis was calculated from four randomly selected regions. Periarterial fibrosis was determined by normalizing the area of PSR stain surrounding an arteriole to arteriolar diameter (diameter = circumference of artery/3.14). Average values for each animal were based on measurements made on four randomly selected coronary arterioles from ZLC (n = 6), ZOC (n = 5), ZOSV (n = 5), ZOV (n = 6) and ZOH (n = 7).

### Analysis of cardiac and aortic 3-nitrotyrosine (3NT)

We evaluated the levels of 3NT as a marker of myocardial nitrosylated oxidation products caused by the formation of peroxynitrite. Five-micron sections of the LV free wall were initially quenched of endogenous peroxidase and incubated overnight with rabbit polyclonal anti-3NT antibody (1:200; Chemicon, Temecula, CA). Sections were washed and incubated with appropriate secondary antibody. Diaminobenzidine (DAB; DAKO) served as a chromogen. Using a 50i Nikon microscope, five randomly selected 10X bright-field images from each section were captured with a CoolSNAP cf camera. Signal intensities of brownish color, which is indicative of the 3NT level, were quantified by MetaVue software. Average values for each group were based on measurements made on four randomly selected coronary arterioles from ZLC (n = 6), ZOC (n = 5), ZOSV (n = 5), ZOV (n = 6) and ZOH (n = 7).

### Analysis of intracardiac cytokine protein profile by Quantibody® Rat Cytokine Array 65

Protein extraction from frozen heart samples was performed by RayBiotech using a proprietary method (Peachtree Corners, GA). Changes in cytokine protein levels in the heart were custom analyzed by RayBiotech using a highly sensitive quantitative ELISA-based Rat Cytokine Array Q67 and evaluated using proprietary software. This array is a combination of 2 nonoverlapping arrays that facilitates quantitative measurement of the concentrations of 67 rat cytokines by using appropriate antibody pairs. Within the array, an individual cytokine was represented four times, along with positive and negative controls which allows for calculating standard deviation. Differences in cytokine expression were expressed as log_2_ fold changes. Unpaired two-tailed t-tests were used to determine the significance (*p* < 0.05) of differentially expressed cytokines between different groups (e.g., ZOC versus ZOSV, ZOV or ZOH). Cytokines that exhibited statistically significant differences between the treatment and control groups were selected for input into Ingenuity Pathway Analysis (IPA, QIAGEN, Germantown, MD) to identify diseases and functions that were affected. Heatmaps were generated using the ggplot2 package for R.

### Statistical analysis

Results are reported as mean ± SE. One-way ANOVA and post hoc *t*-tests (Fisher’s LSD), or corresponding non-parametric Kruskal–Wallis (Dunn’s), as indicated, were performed to examine differences in outcomes between ZL rats and control and treated ZO groups. Alternatively, we performed two-tailed Student *t*-tests between two groups when ANOVA post hoc tests indicated *p* = 0.10. A *p* value < 0.05 was considered significant. Two-way ANOVA was used to compare aortic responses among groups to increasing concentrations of acetylcholine in the tissue bath (group x Ach concentration). Differences in systolic or diastolic blood pressure at the last measurement period following administration of sac/val, val or hydralazine versus untreated ZO rats were determined by Student *t*-tests adjusted by variance. Sample sizes are listed in tables and figures. The heatmaps were normalized by scaling the values using the sum of each row. The z-scores were calculated with the Creative Commons Attribution-ShareAlike 2.0 Generic from the University of Alberta, Canada.

## Results

### Blood pressure and baseline metabolic parameters

**Fig. 1:**
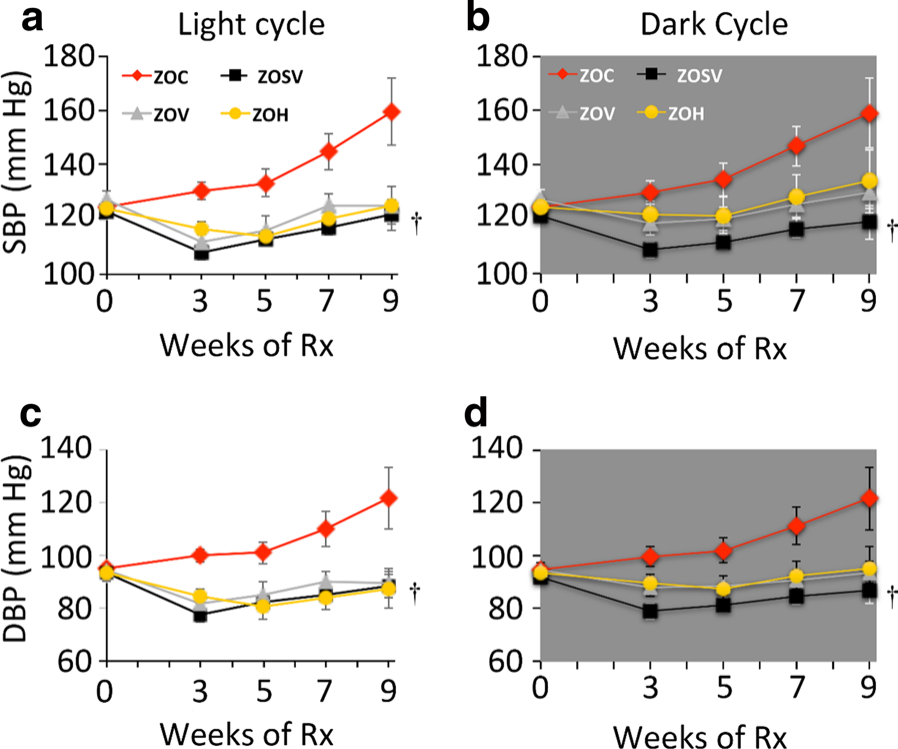
Ambulatory blood pressure was monitored periodically utilizing radio-telemetric transmitters. Systolic blood pressure (SBP) and diastolic blood pressure were recorded prior to the beginning of treatment and after 3, 5, 7 and 9 weeks of treatment during the light and dark cycles. SBP and DBP increased throughout the course of treatment in ZOC. After 9 weeks of treatment SBP and DBP were significantly reduced in ZOSV during the light and dark periods compared to ZOC (*p* < 0.05 indicated by the dagger (^†^) symbol). N = 4, 4, 3 and 4 for ZOC, ZOSV, ZOV and ZOH, respectively

We have previously reported increases in body weight, fat pad mass, fasting glucose, HbA1c, fasting insulin and insulin resistance in ZOC rats and these parameters were unaltered by sac/val, val or hydralazine treatment [16]. The increased systolic and diastolic pressures observed in untreated ZO rats were significantly lowered in all the treatment groups (Fig. 1).

### Structural remodeling and diastolic dysfunction

We have previously reported cardiac hypertrophy and DD in male ZO rats as young as nine weeks of age [23, 24, 28]. Herein, we determined that 10 randomly selected ZOC exhibited cardiac hypertrophy and an abnormal diastolic phenotype prior to initiation of interventions (Table 1), indicating that the study is directed as a treatment strategy rather than a prevention strategy.

At the end of the study period, untreated ZO rats (ZOC) exhibited cardiac hypertrophy evidenced by increases in heart weight (HW) (25%), HW normalized to tibia length (HW/TL) (72%), left ventricular (LV) mass (24%), and anterior wall thickness at end diastole (AWTd) (29%) compared age-matched non-diabetic, Zucker Lean rats (ZLC) (Table 2). ZOC and ZLC exhibited EF ≥ 69% indicating normal systolic function. However, compared to ZLC, ZOC exhibited DD characterized by a decrease in the tissue Doppler derived e’/a’ ratio (38%), in concert with increases in LV filling pressure (E/e’ ratio, 220%), diastolic stiffness (E/e’/LVIDd, 230%), isovolumic relaxation time (IVRT, 47%) and the myocardial performance index (MPI, 23%). Collectively, these parameters demonstrate abnormal LV wall motion during diastole (e’/a’ and E/e’), passive LV wall stiffness (E/e’/LVIDd) and prolongation of the active energy requiring period of relaxation (IVRT). Additionally, DD in ZOC was associated with left atrial remodeling indicated by an increase in the left atrial to aortic root (LA/Ao) ratio (45%), an effect that reflects the response by the thin-walled atrium to increased luminal pressure.

**Table 2:** Ultrasound derived cardiac function and structure parameters of lean and obese Zucker rats

Treatment	ZLC	ZOC	ZOSV	ZOV	ZOH
	(6)	(10)	(11)	(11)	(10)
Heart weight (mg)	1147 ± 41	1429 ± 62*	1392 ± 67*^§^	1302 ± 35*^§^	1639 ± 50*
HW/TL (mg mm^−1^)	10.5 ± 0.3	18.1 ± 0.5*	16.8 ± 0.7*^§^	17.3 ± 0.4^§^	15.8 ± 0.6*
Systolic parameters	(6)	(6)	(6)	(7)	(6)
Ejection fraction (%)	69 ± 3	76 ± 2	81 ± 2*	80 ± 2*	80 ± 4*
Cardiac output (ml min^−1^)	190 ± 23	270 ± 29	281 ± 24*	260 ± 19^§^	342 ± 36*
Stroke volume (µl)	504 ± 52	762 ± 90	828 ± 74*	768 ± 54	874 ± 111*
IVCT (ms)	12.5 ± 0.9	15.5 ± 0.6	11.7 ± 1.3^†^	13.7 ± 1.9	13.5 ± 2.9
s’ (mm sec^−1^)	58 ± 1	42 ± 3*	41 ± 1*	43 ± 3*	48 ± 6
Diastolic parameters	(6)	(6)	(6)	(7)	(6)
E, (mm sec^−1^)	888 ± 56	1117 ± 126	894 ± 53	1095 ± 63	1091 ± 60
A, (mm sec^−1^) late	886 ± 36	1036 ± 127	814 ± 70	973 ± 54	1098 ± 127
E/A ratio	1.00 ± 0.04	1.07 ± 0.08	1.12 ± 0.06	1.15 ± 0.11	1.03 ± 0.11
e’, (mm sec^−1^)	94 ± 7	52 ± 6*	55 ± 7*	56 ± 8*	76 ± 10
a’, (mm sec^−1^)	76 ± 4	69 ± 10	51 ± 3*^§^	76 ± 6^‡^	68 ± 4^‡^
e’/a’ ratio	1.23 ± 0.03	0.76 ± 0.05*	1.08 ± 0.12^†^	0.75 ± 0.08*^‡§^	1.12 ± 0.13^†^
E/e’ (LV filling pressure)	10.3 ± 0.7	22.7 ± 2.6*	17.1 ± 1.5	20.9 ± 2.4*	15.5 ± 2.6
Diastolic stiffness (E/e’/LVIDd)	1.16 ± 0.13	2.68 ± 0.29*	2.09 ± 0.22*	2.51 ± 0.30*^§^	1.67 ± 0.25^†^
IVRT (ms)	17.5 ± 0.7	25.8 ± 1.4*	21.3 ± 0.7*^†^	20.3 ± 0.6*^†^	18.4 ± 1.8^†^
Ejection Time (ms)	64.7 ± 2.4	71.1 ± 1.7	68.4 ± 1.8	66.5 ± 3.1	60.4 ± 2.0
LVETI	702 ± 13	685 ± 17	650 ± 12*	716 ± 32	679 ± 27
MPI	0.47 ± 0.03	0.58 ± 0.01*	0.49 ± 0.03^†^	0.52 ± 0.04	0.52 ± 0.06
Structural parameters	(6)	(6)	(6)	(7)	(6)
LA/Ao ratio	0.95 ± 0.05	1.38 ± 0.11*	1.23 ± 0.10*	1.28 ± 0.03*	1.35 ± 0.05*
LV Mass (mg)	863 ± 35	1067 ± 19*	1104 ± 73*^§^	976 ± 45^§^	1340 ± 64*^†^
AWTd (mm)	1.60 ± 0.07	2.07 ± 0.05*	2.07 ± 0.16*	1.87 ± 0.07	2.03 ± 0.08*
PWTd (mm)	1.68 ± 0.06	1.83 ± 0.11	2.04 ± 0.16*	1.81 ± 0.10	2.08 ± 0.05*
Relative wall thickness	0.38 ± 0.02	0.46 ± 0.03*	0.50 ± 0.04*	0.44 ± 0.02*	0.45 ± 0.02*
LVIDd (mm)	8.71 ± 0.25	8.45 ± 0.21	8.26 ± 0.17^§^	8.32 ± 0.14^§^	9.22 ± 0.26^†^
LVIDs (mm)	5.24 ± 0.29	4.44 ± 0.16*	3.99 ± 0.26*	4.09 ± 0.23*	4.55 ± 0.47

Values are mean ± SE, (sample sizes shown in parentheses). *p < 0.05 vs ZLC; ^†^*p* < 0.05 vs ZOC; ^§^*p* < 0.05 vs ZOH; ^‡^*p* < 0.05 vs ZOL. *HW* Heart weight, *tibia length* tibia length, *LV* left ventricle, *e’* early septal wall velocity during diastole, *a’* late septal wall velocity during diastole, *s’* peak septal wall velocity during systole, *E* early mitral flow velocity, *A* late mitral flow velocity, *E/E’ LV* filling pressure, *IVRT* isovolumic relaxation time, *IVCT* isovolumic contraction time, *MPI* myocardial performance index, *LVETI LV* ejection time index, *LA* left atrium diameter, *Ao* aorta diameter, *AWTd* anterior wall thickness at end diastole, *PWTd* posterior wall thickness at end diastole, *LVIDd LV* inner diameter at end diastole, *LVIDs LV* inner diameter at end systole

**Fig. 2:**
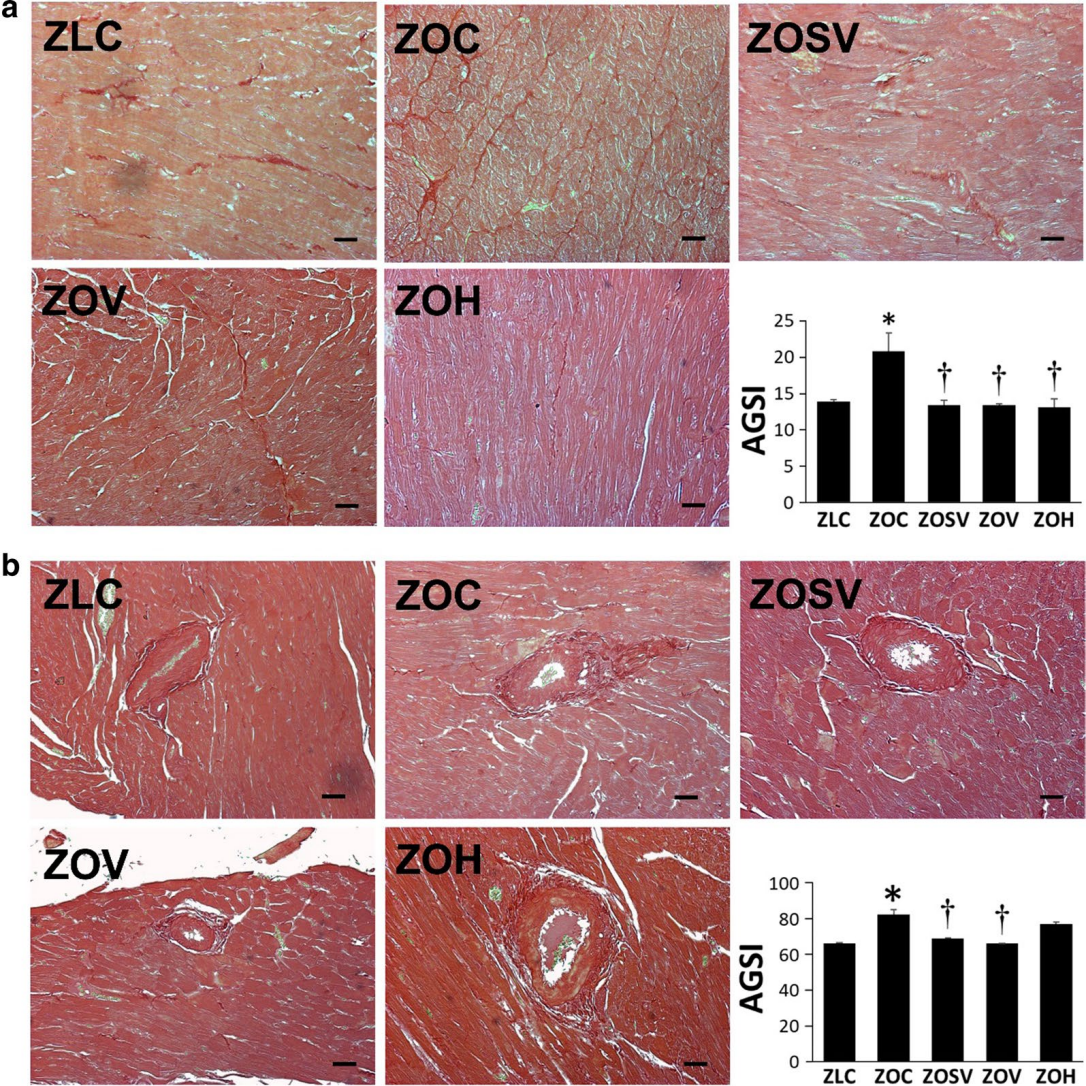
Sacubitril/valsartan (sac/val) reduces left ventricular myocardial interstitial (**a**) and periarterial (**b**) fibrosis in ZO rats. Representative PSR stained images show fibrosis in the myocardial interstitium (**a**) or surrounding an arteriole (**b**). Accompanying bar graphs show quantitative analysis of average intensity of PSR staining in panel **a** and area of fibrosis normalized to arteriole diameter in panel **b**. Data are represented by means ± SE. N = 6, 6, 5, 5 and 7 for ZOC, ZOSV, ZOV and ZOH, respectively. Symbols: *Indicates *p* < 0.05 versus ZLC; ^†^Indicates *p* < 0.05 versus ZOC. Scale bars = 50 µm

Although untreated ZO rats (ZOC) exhibited cardiac hypertrophy indicated by increases in HW, LV mass, and AWTd, these parameters were not altered by sac/val (ZOSV), val alone (ZOV), or hydralazine (ZOH) (Table 2). However, a moderate, but significant, degree of fibrosis in the interstitial region was seen in ZOC compared to ZLC (Fig. 2a). Though sac/val, val and hydralazine were all effective in decreasing fibrosis in ZO rats, the magnitude of decease was not different between the three treated groups. The extent of fibrosis in periarterial region was increased in ZOC rats (Fig. 2b) and was decreased significantly in ZOSV and ZOV, but not in ZOH.

The comparison of different parameters of DD in ZOC and the treated groups is summarized in Table 2. Compared to ZOC, the e’/a’ ratio improved by 42% in ZOSV and 47% in ZOH, but not in ZOV (-1%). Indeed, ZOV was significantly lower than in ZOC, ZOL and ZOH. Compared to ZOC, LV filling pressure (E/e’) was lower by 25, 8 and 32% in ZOSV, ZOV and ZOH, respectively, although the differences were not significant. Similarly, compared to ZOC, diastolic stiffness index (E/e’/LVID) was lower by 22, 6 and 38% in ZOSV, ZOV and ZOH, respectively. IVRT was significantly reduced (improved) by 17, 21 and 29% in ZOSV, ZOV and ZOH, respectively, compared to ZOC. MPI, which is an index of global cardiac function (*i.e*., diastolic and systolic), was improved by 16, 10 and 10% in ZOSV, ZOV and ZOH, respectively, however only ZOSV was significantly lower than ZOC. Sac/val tended to improve diastolic stiffness index and improved e’/a’ (*p* < 0.05) ratio compared to val. Although hydralazine is as effective as sac/val at improving DD parameters, it causes significant increase in LVMI which is considered to contribute to progression of cardiac dysfunction.

### Oxidative stress

**Fig. 3:**
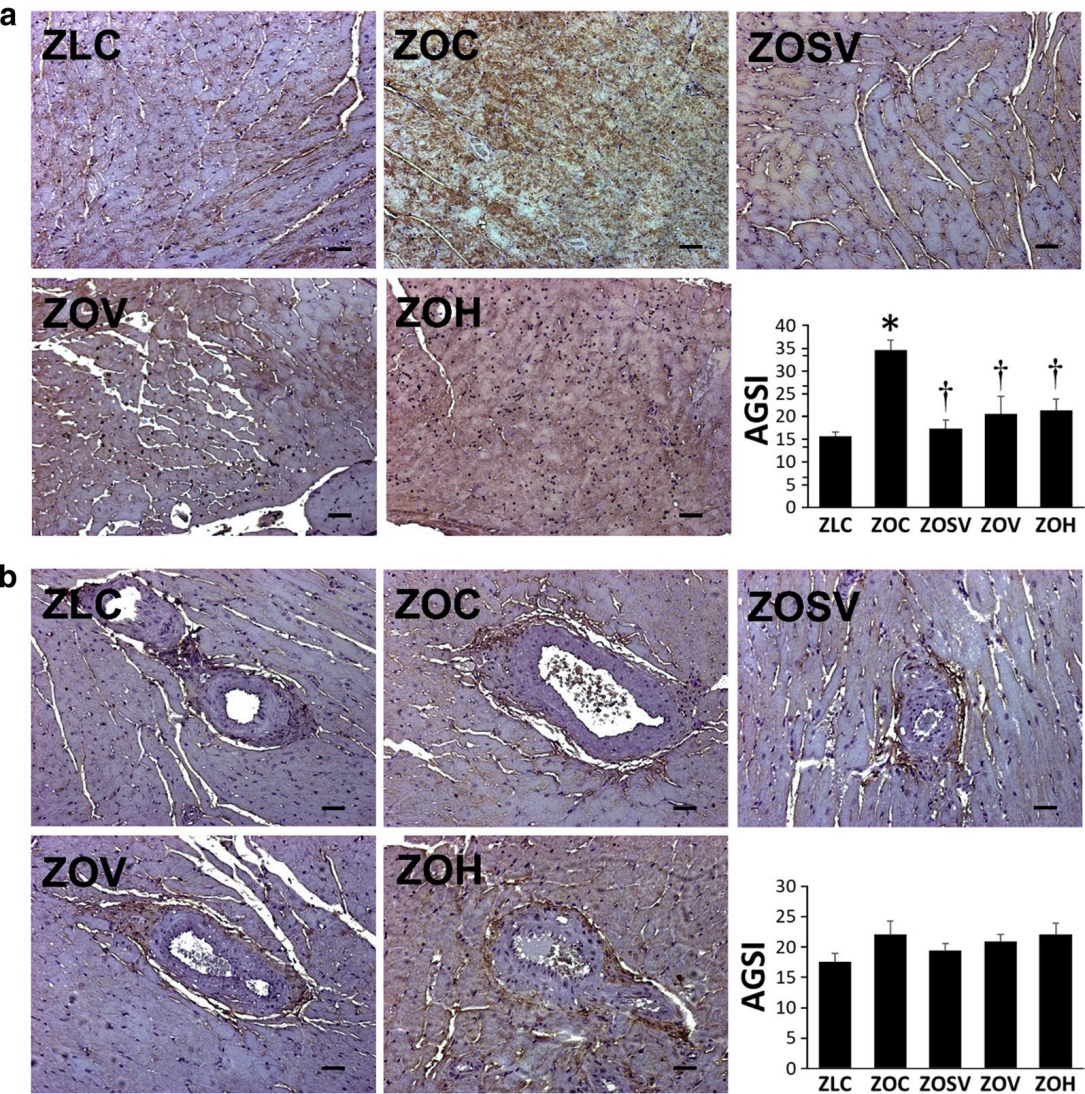
Sacubitril/valsartan (sac/val) reduces myocardial interstitial (**a**) and periarterial (**b**) nitroso- oxidative stress in ZO rats. Representative images of 3-nitrotyrosine immunostaining as a marker for nitroso-oxidative stress with accompanying bar graphs showing quantitation of measures of intensity. Data are represented by means ± SE. N = 5, 5, 5, 5 and 5 for ZOC, ZOSV, ZOV and ZOH, respectively. Symbols: *Indicates *p* < 0.05 versus ZLC; †Indicates *p* < 0.05 versus ZOC

Oxidative stress is an important determinant that either precedes or is associated with the development of DD in rodent models of obesity-associated cardiomyopathy [26, 30]. We have evaluated oxidative stress by analyzing the extent of 3-nitrotyrosine (3NT) accumulation in both interstitial and periarterial regions in the heart. Accumulation of 3NT in interstitium is increased in untreated ZO rats (*p* < 0.05 vs ZLC), but decreased in all three treatment groups (Fig. 3a) (*p* < 0.05 versus ZOC). In contrast to interstitial 3NT accumulation, periarterial 3NT was marginally increased in ZO rats compared to control ZLC rats (*p* = 0.06), and neither sac/val, val or hydralazine had a significant effect on periarterial nitroso-oxidative stress (Fig. 3b).

### Aortic stiffness and relaxation

**Fig. 4:**
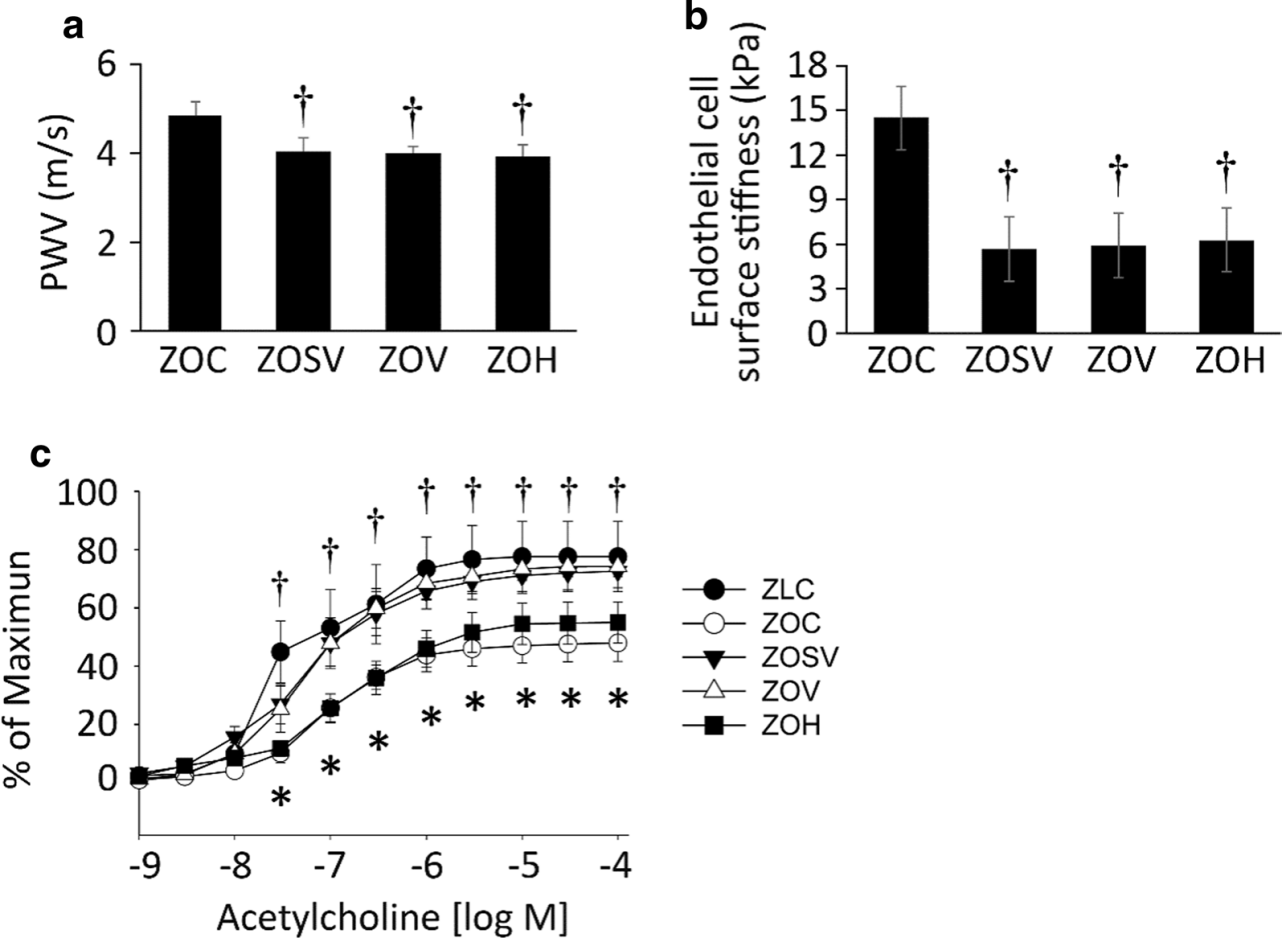
Sacubitril/valsartan (sac/val) ameliorates in vivo aortic stiffening, as well as endothelial stiffening in ex vivo aortic explants. **a** Pulse wave velocity (PWV) measured after 10 weeks of treatment. N = 6, 5, 5, and 7 for ZOC, ZOSV, ZOV and ZOH, respectively. **b** Force measurements were acquired by interaction between a cantilever tip and the EC surface of aortic explants from rats after 10 weeks of treatment. N = 4, 4, 4, 5 and 3 for ZOC, ZOSV, ZOV and ZOH, respectively. **c** Sac/val (▼) and val (○) treatments prevent impaired responses to the nitric oxide dependent vasodilator, acetylcholine in aortic rings of ZO rats (○). Note the normal reactivity of in ZLC aortae (●). Data are represented by means ± SE in the accompanying bar graph. N = 3, 8, 8, 7 and 7 for ZLC, ZOC, ZOSV, ZOV and ZOH, respectively. Symbols: *Indicates *p* < 0.05 versus ZLC; ^†^Indicates *p* < 0.05 versus ZOC; indicates ^§^*p* < 0.05 versus ZOH

We have determined aortic compliance by measuring in vivo pulse wave velocity (PWV) and ex vivo aortic stiffness by atomic force microscopy (AFM) in aortic explants. Compared to untreated ZO rats, PWV was decreased similarly in all three treatment groups (*p* < 0.05) (Fig. 4a). Endothelial cell surface stiffness was also significantly decreased in ZOSV, ZOV and ZOH groups compared to the ZOC group (Fig. 4b). We further examined endothelial dependent aortic relaxation. We previously reported impaired endothelium-dependent vasodilation in younger and older ZO rats [24, 26]. We anticipated impaired endothelium-dependent vasodilatory responses to acetylcholine and insulin in the aorta of ZOC and improvement with sac/val, to a greater extent than to val or hydralazine. As expected, acetylcholine-induced vasodilation was significantly lower in ZOC relative to ZLC (Fig. 4c). However, treatment with sac/val or val, but not hydralazine, showed normal responses to acetylcholine, and are similar to ZLC. Responses to insulin and the endothelium-independent vasodilator, sodium nitroprusside were not different among the groups tested (not shown).

### Cytokine measurements

**Fig. 5:**
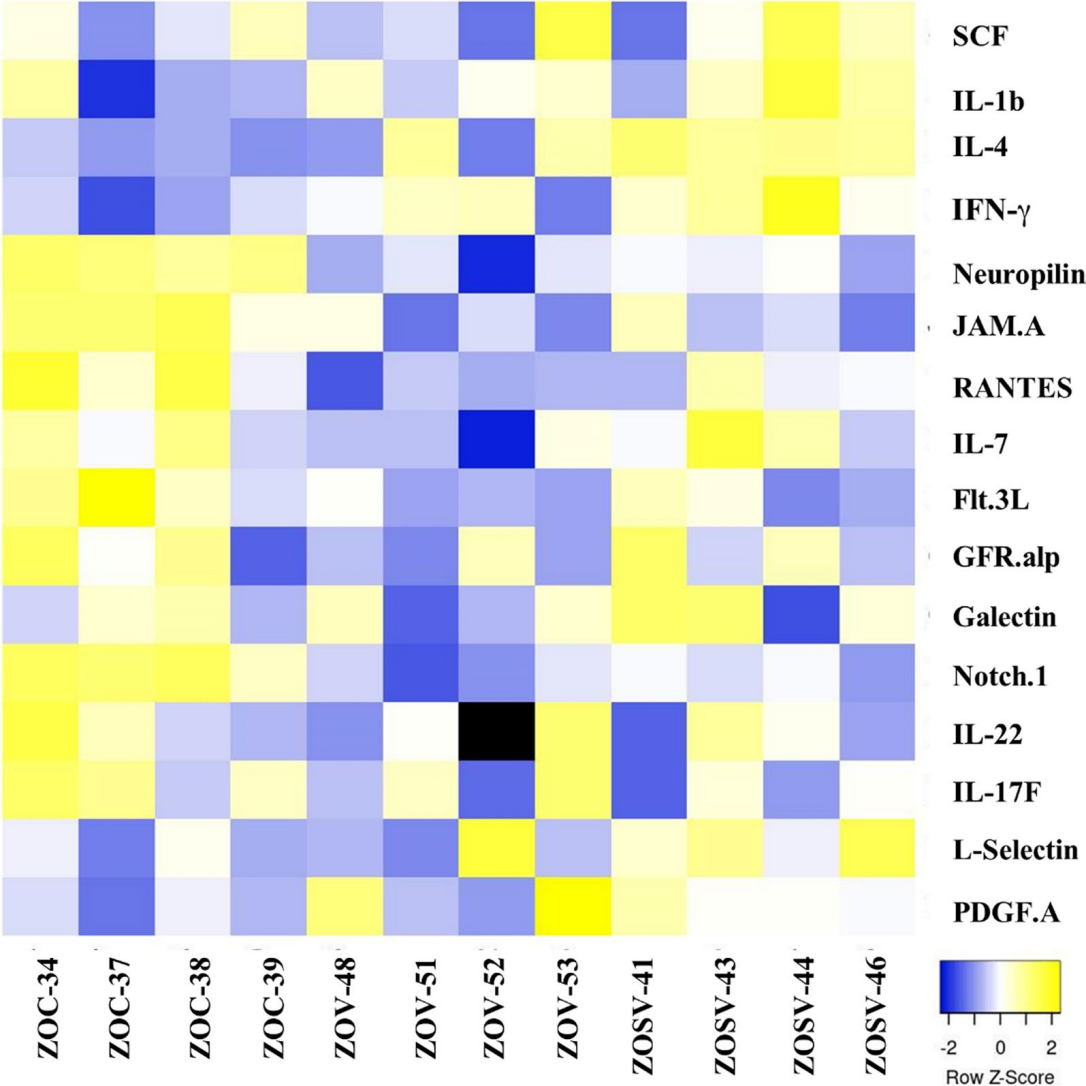
Heatmap illustrating differential cytokine expression in ZOC, ZOV and ZOSV rat cardiac lysates (N = 4 per group). Individual samples are shown on the x-axis. The y-axis shows statistically significant (*P* ≤ 0.05), differentially expressed cytokine markers among the three groups. Each row in the heatmap represents relative changes in the normalized protein expression in the control and treated rats. Need acronyms for cytokines. *SCF* stem cell factor, *IFN-γ* interferon gamma, *JAM.A* junctional adhesion molecule-A, *RANTES* regulated on activation, normal T cell expressed and secreted, *Flt.3L* Fms-like tyrosine kinase 3 ligand; GFR-alpha-1, *PDGF-A* platelet derived growth factor-A

To explore the molecular mechanisms in the ZLC, ZOC, ZOSV, and ZOV groups (N = 4 in each group) that could potentially mediate the functional/structural changes in the heart, we used a commercial service to measure 67 cytokines using a Cytokine Array. Given the exploratory nature of these experiments and the small sample size, we did not adjust the significance values for multiple testing and p-value ≤ 0.05 was considered significant. We found that five cytokines, namely RANTES, PDGF-AA, GFR-alpha-1, SCF, and IL-7, were differentially expressed between the lean (ZLC) and obese (ZOC) rats (Table 3). Relative to the ZOC rats, the ZOV rats showed downregulation of five proteins, namely, Neuropilin-1, Notch-1, JAM-A, RANTES, and Flt-3L (Table 4). Remarkably, the ZOSV rats showed reduced expression of Neuropilin-1, Notch-1, and JAM-A relative to ZOC rats which was similar to that seen in ZOV rats (Table 4). In addition, the ZOSV rats showed increased expression of PDGF-AA, L-Selectin, IFN-γ, and IL-4 when compared to the ZOC rats. A heatmap of the relative differences of statistically significant cytokines in cardiac lysates across the ZOC, ZOV, and ZOSV rats is shown in Fig. 5. The increased expression of the aforementioned four proteins distinguishes ZOSV rats from ZOV rats suggesting that they could potentially mediate the effects associated with sac/val treatment.

**Table 3:** Summary of cytokine profile changes in Zucker Obese (ZOC) rats when compared to control Zucker Lean rats (ZLC)

Molecule	Gene	ZOC/ZLC	
		log_2_FC	*P*-value
RANTES	*Ccl5*	− 1.288	0.050
PDGF-AA	*Pdgfa*	− 1.359	0.012
GFR-alpha-1	*Gfra1*	− 0.885	0.049
SCF	*Kitl*	− 0.269	0.043
IL-7	*Il7*	− 0.655	0.024

*RANTES* Regulated upon Activation, Normal T Cell Expressed and Presumably Secreted, *PDGF* Platelet Derived Growth Factor, *GFR-alpha-1* Glial Cell line-derived Neurotrophic Factor Receptor Alpha 1, *SCF* Stem Cell Factor, *IL* Interleukin

Molecules on the cytokine array that showed at least log_2_ fold-change (log_2_FC) of 1.2 and with a *p*-value ≤ 0.05 are shown. Statistical significance between the indicated groups determined by unpaired two-tailed *t*-tests. Gene symbols corresponding to each cytokine are shown. Sample size: N = 4/group

**Table 4:** Summary of cytokine profile changes in Zucker Obese (ZO) rats treated with sacubitril/valsartan (ZOSV), valsartan (ZOV), and hydralazine (ZOH) when compared to control rats (ZOC)

Molecule	Gene	ZOSV /ZOC		ZOV/ZOC		ZOH/ZOC	
		log_2_FC	P-value	log_2_FC	P-value	log_2_FC	P-value
Neuropilin-1	*Nrp1*	− 0.266	0.001	− 0.398	0.005	− 0.173	0.052
Notch1	*Notch1*	− 0.768	0.003	− 1.108	0.002	− 0.976	0.002
PDGF-AA	*Pdgfa*	1.054	0.041				
L-Selectin	*Sell*	0.800	0.044				
IFNg	*Ifng*	0.550	0.020				
IL-4	*Il4*	0.386	2.61E-05				
JAM-A	*F11r*	-− 0.271	0.021	− 0.317	0.010		
RANTES	*Ccl5*			− 0.436	0.014		
Flt-3L	*Flt3l*			− 0.464	0.047		
IL-17F	*Il17f*					− 0.668	0.037
Galectin-3	*Lgals3*					− 0.572	0.044

*Notch* Notch receptor 1, *IFN* Interferon, *JAM-A* Junctional Adhesion Molecule A, *Flt-3L* FMS related Receptor Tyrosine Kinase 3 Ligand

Molecules on the cytokine array that showed at least log_2_ fold-change (log_2_FC) of 1.2 and with a *p*-value ≤ 0.05 are shown. Statistical significance between the indicated groups determined by unpaired two-tailed *t*-tests. Gene symbols corresponding to each cytokine are shown. Sample size: N = 4/group

### IPA analysis

**Fig. 6:**
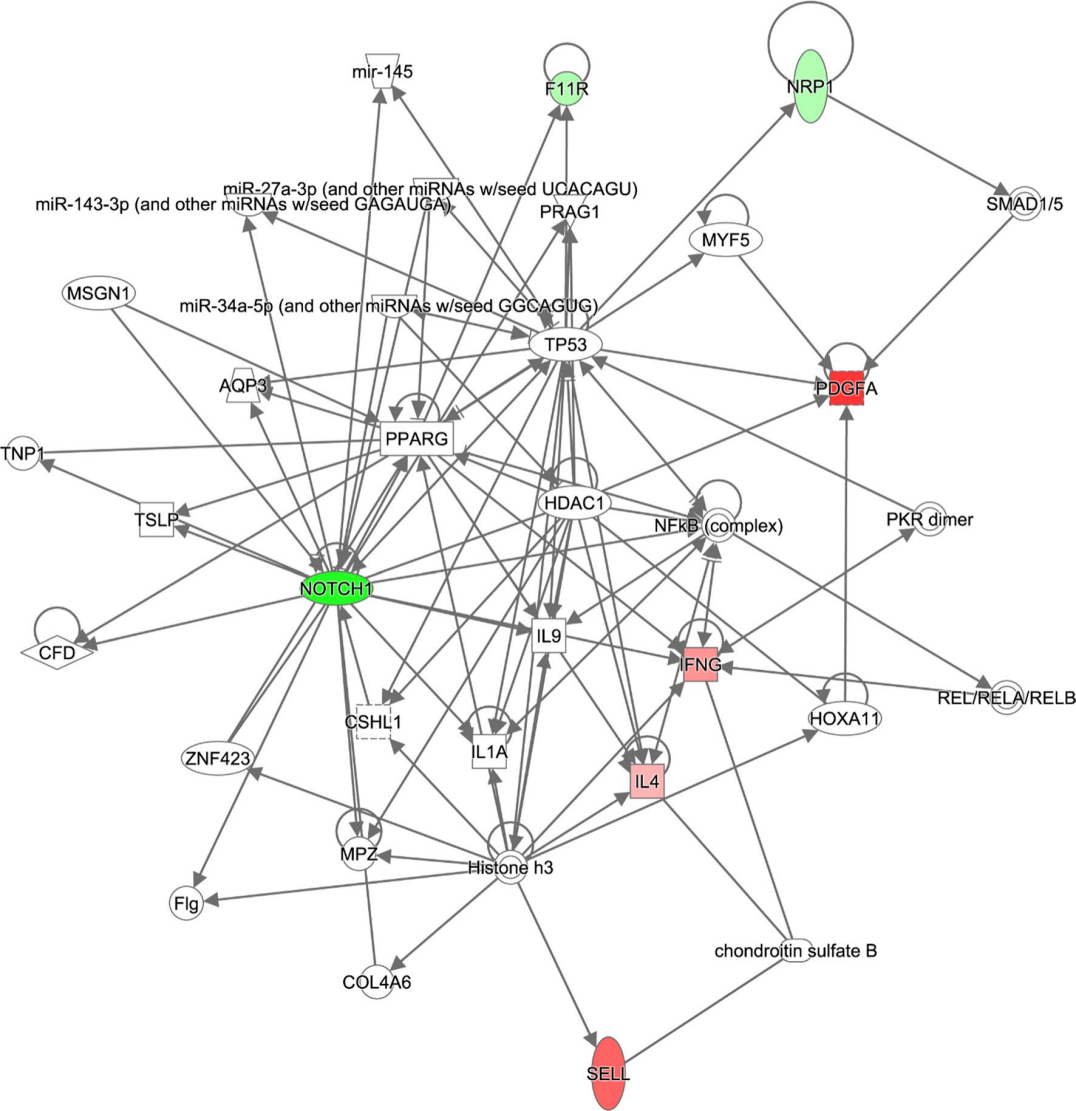
Network created by Ingenuity Pathway Analysis from the ZOSV rats. The network was generated from differentially expressed proteins when compared to ZOC rats (N = 4 per group). The list was selected based on the proteins meeting the threshold of log_2_FC of 1.2 and *p*-value ≤ 0.05. Colored nodes represent the genes derived from our uploaded protein list (focus genes) with green nodes representing downregulation and red nodes representing upregulation. White nodes represent genes or molecules that could potentially be connected to the focus genes and are derived from the IPA knowledge base. The solid lines represent direct interactions between the nodes. A detailed explanation of the molecule shapes is available at http://qiagen.force.com/KnowledgeBase/articles/Basic_Technical_Q_A/Legend

We then sought to map the differentially expressed cytokines detected in the ZOL rats to the networks that may mediate the effects of sac/val that are accessible through the Ingenuity database. We did not perform a similar analysis for the ZOV rats as only five cytokines were differentially expressed in these rats relative to ZOC and three of them were also present in the ZOL rats. Our analysis generated a single network with a score of 21 with the following associated functions: cellular development, cellular movement, cellular growth, and proliferation (Fig. 6). The network included the following focus (experimentally detected) molecules: NOTCH-1, IL-4, IFN-γ, PDGFA, F11R, L-Selectin (SELL), and NRP1. Remarkably, one of the focus molecules of the network is Notch-1, a key player in vascular remodeling and endothelial and SMC communication. The cellular and molecular functions of the molecules in the network were related to cell morphology, cell movement, cell death and survival. cell-to-cell signaling and interaction, and cellular development. The top disease and functional annotation of these molecules is the inflammatory response (P = 2.54E−04–1.79E−10) and are related to macrophage polarization (*P* = 1.79 E−10) and leukocyte emigration (*P* = 1.07E−9). Of note, mapping these molecules to clinical pathology endpoints suggested an association with cardiac enlargement (*P* = 6.41E−02–6.20E−05). The top canonical pathways predicted were the Th1 (*P* = 5.07E−06) and Th2 pathways (*P* = 7.20E−06). Notably, IL-4 that anchors Th2 pathway plays a key role in macrophage polarization that is important in vascular remodeling.

## Discussion

In the present investigation, we determined the potential therapeutic effects of sacubitril/valsartan (sac/val; LCZ696), a combination of Ang II receptor blocker and neprilysin (NEP) inhibitor (ARNi), on cardiovascular remodeling and function in pre-diabetic obese rats. We then compared the effects of dual blockade (sac/val) with val monotherapy, and to those of hydralazine, a blood pressure lowering drug, to determine the cardiovascular protection. Furthermore, we performed cytokine array analysis of selected proteins showing differences in distinct signaling pathways. For the first time, we have shown the enhanced cardiovascular protection afforded by sac/val on selective parameters of diastolic dysfunction, as well systolic dysfunction, and large artery stiffness compared to val and hydralazine.

Diastolic dysfunction (DD), characterized by impaired relaxation of the left ventricle, is one of the early manifestations of heart failure with preserved ejection fraction (HFpEF) [31, 32]. Childhood-adolescent overweight and obesity are major health burdens around the globe and the presence of DD has significant impact as a risk factor for the progression of CVD [4, 5]. However, drugs targeting systolic dysfunction are not usually considered as effective in the management of DD, thereby necessitating the need to develop drugs that specifically target DD, especially in the setting of obesity [33, 34]. In this regard, obesity-induced DD is associated with multiple abnormalities, including inappropriate activation of tissue Renin–Angiotensin–Aldosterone System (RAAS) [24, 35, 36], as well as, normal or subnormal responses to ANP in the early stages of the disease or enhanced ANP responsiveness at later stages [17]. Sac/val is a first-in-class approved ARNi, that simultaneously provides Ang II type I receptor blockade and NP inhibition. Cardiac protection afforded by sac/val is well recognized with completion of first and second phase clinical trials [37–41]. A recent study also favored the beneficial effects of sac/val in HFpEF patients [42]. Therefore, we have chosen Zucker Obese rats, a preclinical model of early DD, to represent young patients with cardiometabolic syndrome with a constellation of features, including hypertension, hyperlipidemia and insulin resistance [43]. We then compared the efficacy of sac/val with val alone and with hydralazine, a blood pressure lowering agent.

All three drugs, sac/val, val and hydralazine, improved IVRT, a parameter of prolongation of the active energy phase of relaxation. However, impairment in LV wall motion during diastole, observed in untreated ZOC rats and indicated by an increase in the e’/a’ ratio, was improved by sac/val, but not val. Moreover, sac/val, but not val, tended to improve passive LV wall stiffness (E/e’/LVIDd). MPI (Myocardial Performance Index), which is an index of global cardiac function (*i.e*., diastolic and systolic), was significantly improved only by sac/val, potentially due in part to improvement in IVCT. Taken together, sac/val treatment appears to be superior in improving DD in this preclinical model of obesity cardiomyopathy. Interestingly, the enhanced efficacy of sac/val occurred without significant improvement in blood glucose or insulin levels and suggests that these benefits occur independent of glycemic control.

The factors contributing to HFpEF in contrast to HFrEF have been diverse, including endothelial dysfunction, changes in intrinsic cardiomyocyte stiffness with relaxation abnormalities, LV hypertrophy, and low-grade metabolic inflammation with variable extent of fibrosis and oxidative stress [44–47]. Moreover, both association and dissociation of Ang II and the NP system with markers of oxidative stress, blood pressure, maladaptive immune and inflammatory response, cardiac hypertrophy and fibrosis have all been reported in the context of preclinical DD and HF resulting from mechanical overload, genetic models of leptin resistance, and inappropriate activation of the RAAS [22, 24, 48–50]. Cardiac fibrosis, intrinsic stiffness and hypertrophy have all been shown to be caused, in part, by enhanced ROS generation in the cardiovascular tissue [9, 51, 52]. In fact, we have examined all of these components in the present study. Although we found suppression of ROS (3-NT) in LV interstitium by all three drugs to a similar extent, suggesting that suppression of oxidative stress may, in part, contribute to improvement in DD, and the beneficial effects of sac/val may be mediated by factors independent of ROS accumulation.

Recent advances in gene expression and multiplexed protein arrays are increasingly used to understand the molecular pathophysiology of DD and HF in human and animal models of obesity, diabetes, pressure overload and myocardial infarction [53–55]. In the ZO heart tissue the increases in cytokines, such as IL-6 and TNF-α are only marginally increased [56]. In the mouse model of diet-induced obesity (DIO), IL-6 levels are either not significantly altered or increased significantly depending on the dietary components [57, 58]. However, in a recent study investigating the effect of sac/val in a DIO model, the impact of this drug combination on cytokines was not investigated [22]. Therefore, we analyzed changes in multiple cytokines using a cytokine array. In the current investigation, the array analysis revealed, among other things, that Notch-1 is markedly decreased by both sac/val and val. However, it appears that the effects of Notch-1 are cell type-specific. For example, Notch-1 exerts protective effects in endothelial cells and cardiomyocytes, but plays a deleterious role in fibroblasts and immune cells [59, 60]. Interestingly, both ZOSV and ZOV showed improvement in DD and suppressed Notch-1 expression, suggesting that the beneficial effects of sac/val and val in the ZO model of obesity-associated DD may be, in part, explained by suppression of Notch-1 in fibroblasts and immune cells. However, ZOSV showed increased expression of IFN-γ compared to the other groups. Although IFN-γ is implicated in immune and inflammatory responses as a component of the Th1 response, its increased expression has been shown to exert contrasting effects; promote cardiovascular dysfunction or mediate cardioprotective effects [61–66]. However, unlike in other studies [62–66], changes in IFN-γ levels did not correlate with suppression of cardiac hypertrophy in the sac/val-treated ZO rats.

IL-4 levels were also elevated in ZOSV, but not in ZOV, suggesting that increased IL-4 levels in this treated group might have contributed to the higher impact of sac/val over val in ZO rats. In fact, IL-4 is emerging as a cardioprotective and repurposing molecule to improve cardiac dysfunction [67, 68]. IL-4 signals via IL-4Rα/IL-13Rα1, a hybrid receptor used by both IL-4 and IL-13. It is noteworthy that the expression of this hybrid receptor is impaired in HF patients [69, 70]. Although IL-4 does not suppress cardiac fibrosis, it enhances cardiac function by improving cardiac metabolic functions possibly through signaling via this hybrid receptor to promote M2 macrophage polarization and suppression of cardiac inflammatory response [67–70]. Therefore, it is plausible that upregulation in IL-4 signaling in the sac/val treated ZO rats might have contributed to further improvements in DD compared to val alone. The cytokine array also revealed that RANTES expression is suppressed in untreated and all treated groups, and more so in the ZOV group. In this regard, hyperinsulinemia as seen in ZO rats might have contributed to suppression of RANTES, although its role in DD is not fully understood [71, 72]. However, a limitation of our cross-sectional study design is that it does not capture the dynamic changes in the cytokine profile over the treatment period. In the future, we will include a longitudinal study design to gain further mechanistic insights into the beneficial effects of sac/val treatment.

Two critical determinants of cardiovascular protection are bioavailable nitric oxide and cyclic GMP [73]. While Ang II could decrease bioavailable nitric oxide by causing insulin-mediated impairment of eNOS activation and nitric oxide destruction, an increase in cyclic GMP by ANP will further enhance accumulation of cyclic GMP with a concomitant increase in nitric oxide production and signaling [57]. Therefore, additional provision of cyclic GMP by sac/val compared to val alone might have contributed to further improvements in diastolic function observed in this study. Additional risk factors like hypertension and myocardial hypertrophy also contribute to DD. Surprisingly, all three drugs decreased blood pressure to a similar extent. The superior cardioprotective effects afforded by sac/val compared to val in improving DD, despite similar BP responses in the two groups, suggests there may be a BP independent component to improvement in DD with the addition of sacubitril. Moreover, we did not find improvements in structural abnormalities, including cardiac hypertrophy, by sac/val or val, suggesting further that improvement in diastolic function occurs independent of improvements in structural remodeling. In fact, improvement in diastolic function, independent of changes in blood pressure, hypertrophy and structural remodeling, has been reported in both preclinical models of obesity cardiomyopathy and human patients [26, 74].

Obesity and diabetes affect the cardiovascular system by indirect and direct mechanisms involving both vascular and myocardial disorders, respectively. In this regard, improvement in aortic stiffness is often associated with better vascular health, and in turn diastolic function. Sac/val (ARNi) therapy has recently been shown to improve aortic compliance in HF patients [75]. In concordance, our results also show that sac/val significantly improved aortic compliance by decreasing aortic stiffness, as evaluated by PWV. Although arterial stiffness increases naturally with aging, the process is accelerated and occurs prematurely in the setting of obesity, insulin resistance and diabetes [7]. Increased systolic pressure and decreased diastolic pressure result in increased pulse pressure caused by stiffening of central arteries. An increase in systolic pressure causes an increase in cardiac afterload, left ventricular mass, and oxygen demand, whereas decreased diastolic pressure impairs coronary blood flow. These changes can result in left ventricular remodeling, ischemia and fibrosis, all of which contribute to left ventricular diastolic dysfunction and coronary heart disease [7].

We used acetylcholine-dependent aortic relaxation as a surrogate for nitric oxide-dependent endothelial function. We observed improved acetylcholine-dependent vasorelaxation by sac/val and val. However, no such improvement was seen with hydralazine. PWV and endothelial surface stiffness measure overall stiffness of the vascular wall of aorta which was decreased by hydralazine, suggesting dissociation between vascular stiffness and nitric oxide-dependent vasodilatation. We have previously reported impaired microvascular remodeling as shown by increased Renal Resistivity Index (RRI) and renovascular fibrosis by hydralazine, but not sac/val and val [16]. In this study, we also observed perivascular fibrosis in the hydralazine treated ZO rats along with enhanced LV mass suggesting increased strain on the part of ventricles, in part, due to microvascular stiffening by hydralazine. In this regard, it is noteworthy that although hydralazine is used in the management of hypertension, it is also known to suppress acetylcholine-induced vasorelaxation, as observed in the present study [76].

Our study has some limitations. First, we have not measured cardiac or systemic ANP or BNP levels. In this regard, increased levels of NPs have been reported in symptomatic HF subjects, and are considered an indication of myocardial response to hemodynamic alterations or impaired responsiveness to the effects of NP due to counter regulatory signaling pathways [77–79]. In contrast, NP levels are often lower in the setting of obesity due to either impaired NP production or its enhanced degradation [80, 81]. Second, we used ZO rats that are considered somewhat older (26 weeks) compared to the studies performed in younger animals. However, we have evaluated renal protection by these three drugs in a companion paper [16] and renal injury is usually manifested a few weeks later than the earliest manifestation of DD in these ZO rats [56, 82].

## Conclusion

**Fig. 7:**
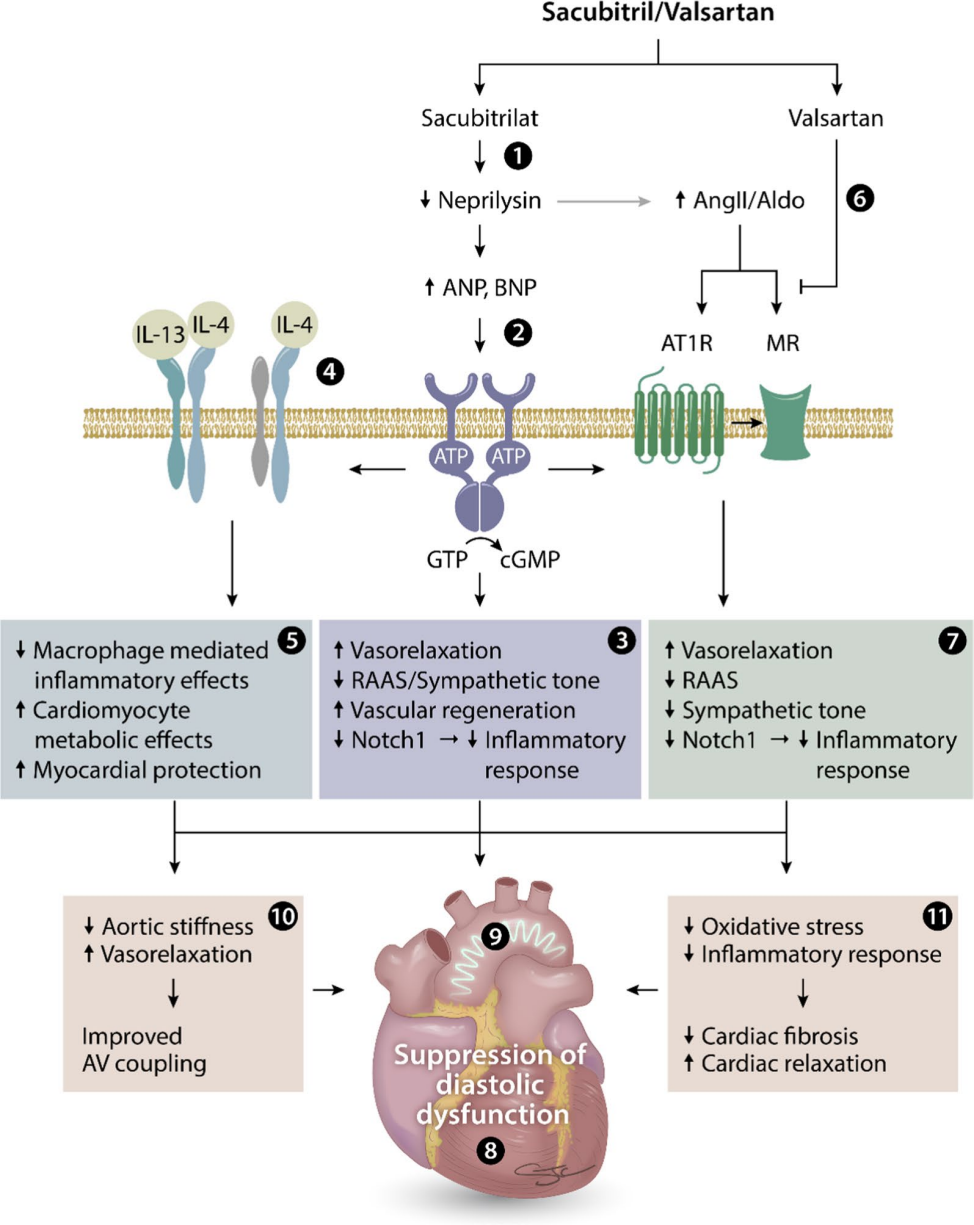
Schematic showing the potential beneficial effects of sacubitril/valsartan combination in a genetic model of obesity and early diabetes with established diastolic dysfunction. The prodrug sacubitril is converted to sacubitrilat (LBQ657) in vivo via de-ethylation by esterases, and is a known inhibitor of neprilysin and ANP and BNP degradation (1). By enhancing ANP and BNP levels and subsequent conversion of GTP to cGMP (2), sacubitrilat improves vasorelaxation and vascular regeneration and decreases RAAS/sympathetic tone and Notch1-dependent inflammatory signaling (3). Additional enhancement of IL-4 receptor signaling by sacubitrilat (4), but not valsartan, suppresses macrophage-mediated inflammatory responses and enhances metabolic functions in cardiomyocytes through an IL-4/IL-13 hybrid receptor (5). Valsartan, on the other hand, blocks AT1R, and decreases aldosterone levels and MR signaling (6), resulting in improved vascular relaxation and decreased RAAS, sympathetic tone and Notch1-dependent inflammatory responses (7). Collectively, this drug combination reduces diastolic dysfunction (8) and vascular stiffness (9) by reducing aortic stiffness, oxidative stress, inflammatory responses, and cardiac fibrosis (10 and 11) by modulating ANP/BNP, AT1R, MR and IL-4/IL-13 mediated signaling in a genetic model of obesity and early diabetes. ANP, Atrial Natriuretic Peptide; AngII, Angiotensin II; Aldo, Aldosterone; BNP, Brain Type Natriuretic peptide; AT1R, Angiotensin type 1 Receptor; MR, Mineralocorticoid Receptor; IL-4, Interleukin-4; IL-13, Interleukin-13; ATP, Adenosine Triphosphate; GTP, Guanosine triphosphate; cGMP, cyclic guanosine monophosphate; RAAS: Renin–Angiotensin–Aldosterone System; Notch1, Notch homolog 1 translocation-associated (drosophila)

The prognosis of patients diagnosed with heart failure with preserved ejection fraction (HFpEF) remains poor, specifically in patients with comorbid conditions like obesity and diabetes. Further, there are no evidence-based therapies to treat HFpEF. Therefore, therapeutic strategies are urgently needed to improve cardiovascular outcomes in subjects with HFpEF, including diastolic dysfunction [10]. Using a preclinical model of obesity and pre-diabetes, here we report that sacubitril/valsartan (sac/val; LCZ696), a combination of Ang II receptor blocker and neprilysin (NEP) inhibitor (ARNi), improved echocardiographic parameters of ventricular stiffness that were impaired in untreated ZO rats. Moreover, sac/val was more effective at ameliorating diastolic dysfunction compared to val alone. Sac/val also blunted elevated vascular stiffness in ZO rats more effectively than val monotherapy. Although the antihypertensive drug hydralazine was as effective as sac/val in improving parameters of diastolic function, it failed to affect periarterial fibrosis and aortic compliance. While the immune suppressive effects of sac/val treatment are well recognized, our cytokine array analysis also suggests that this drug combination can have protective effects on DD through a previously unrecognized effect on IL-4 induction (Fig. 7). Thus, sac/val treatment may contribute to improvement in cardiovascular stiffness through both common and novel pathways involving immune suppression and tissue repair. Our results also show that the sac/val combination improved diastolic function better than val monotherapy, suggesting a role for enhanced cardiac cyclic GMP signaling. Taken together, these studies suggest that sac/val is superior to val in reversing obesity-associated DD, and is an attractive drug combination for the treatment of early asymptomatic diastolic dysfunction in obesity and prevention of progression of CVD leading to HFpEF.

## Data Availability

The datasets used and/or analyzed during the current study are available from the corresponding author on reasonable request.

## Abbreviations

ACEi: ACE inhibitor
AFM: Atomic Force Microscopy
ARB: Angiotensin type 1 Receptor (AT1R) Blocker
ANP: Atrial Natriuretic Peptide
ARNI: Angiotensin Receptor-Neprilysin Inhibition
cGMP: Cyclic Guanosine MonoPhosphate
CV: Cardiovascular
CVD: CV Disease
DAB: Diaminobenzidine
DBP: Diastolic Blood Pressure
DD: Diastolic Dysfunction
DIO: Diet-induced Obesity
DPP-4: Dipeptidyl Peptidase-4
ECG: Electrocardiogram
EF: Ejection Fraction
HFpEF: Heart Failure with preserved Ejection Fraction
Flt.3L: Fms-like tyrosine kinase 3 ligand
GFR-alpha-1: Glial Cell Line-derived Neurotrophic Factor Receptor Alpha-1
HFrEF: Heart Failure with reduced Ejection Fraction
IFN-γ: Interferon gamma
IL: Interleukin
IL-4Rα: Interleukin-4 receptor subunit alpha
IL-13Rα1: IL-13 receptor subunit alpha 1
IVRT: Isovolumic relaxation time
JAM.A: Junctional adhesion molecule-A
MR: Mineralocorticoid receptor
3-NT: 3-Nitrotyrosine
LV: Left ventricle
NP: Natriuretic peptide
NEPi: Neprilysin inhibition
eNOS: Endothelial nitric oxide synthase
Notch-1: Notch homolog 1 translocation-associated (drosophila)
PSR: Picrosirius red
PDGF-AA: Platelet Derived Growth Factor-AA
PWV: Pulse wave velocity
RANTES: Regulated upon Activation, Normal T Cell Expressed and Presumably Secreted
RRI: Renal Resistivity Index
sac: Sacubitril
val: Valsartan
Sac/val: Sacubitril/valsartan
RAAS: Renin–Angiotensin–Aldosterone System
SGLT-2: Sodium-Glucose coTransporter-2
SCF: Stem cell factor
SBP: Systolic Blood Pressure
T2DM: Type 2 Diabetes Mellitus
ZLC: Zucker Lean Control
ZOC: Zucker Obese Control
ZOSV: Zucker Obese sac/val
ZOV: Zucker Obese valsartan
ZOH: Zucker Obese hydralazine
